# An exact test to detect geographic aggregations of events

**DOI:** 10.1186/1476-072X-9-28

**Published:** 2010-06-07

**Authors:** Rhonda J Rosychuk, Jason L Stuber

**Affiliations:** 1 Department of Pediatrics, 11402 University Avenue NW, Edmonton, Alberta, Canada; 2 Women and Children's Health Research Institute, Edmonton, Alberta, Canada; 3 Department of Chemistry, 200 University Avenue West, Waterloo, Ontario, Canada

## Abstract

**Background:**

Traditional approaches to statistical disease cluster detection focus on the identification of geographic areas with high numbers of incident or prevalent cases of disease. Events related to disease may be more appropriate for analysis than disease cases in some contexts. Multiple events related to disease may be possible for each disease case and the repeated nature of events needs to be incorporated in cluster detection tests.

**Results:**

We provide a new approach for the detection of aggregations of events by testing individual administrative areas that may be combined with their nearest neighbours. This approach is based on the exact probabilities for the numbers of events in a tested geographic area. The test is analogous to the cluster detection test given by Besag and Newell and does not require the distributional assumptions of a similar test proposed by Rosychuk et al. Our method incorporates diverse population sizes and population distributions that can differ by important strata. Monte Carlo simulations help assess the overall number of clusters identified. The population and events for each area as well as a nearest neighbour spatial relationship are required. We also provide an alternative test applicable to situations when only the aggregate number of events, and not the number of events per individual, are known. The methodology is illustrated on administrative data of presentations to emergency departments.

**Conclusions:**

We provide a new method for the detection of aggregations of events that does not rely on distributional assumptions and performs well.

## Background

In disease surveillance, statistical methods can be used to identify geographical areas that have statistically higher numbers of cases of disease than expected by chance. These geographical areas with aggregations of disease are called clusters. In some situations, the disease incidence or prevalence may not be the most or only relevant feature for analysis, and the analysis of events related to diseased individuals may be more appropriate. For the delivery of health services through emergency departments (EDs), the number of presentations to EDs can be more relevant than the number of distinct individuals seen in the ED. If there are many individuals that have multiple presentations, analysis based solely on the number of individuals, and not the number of presentations, will only be able to identify clusters with excess numbers of individuals and not clusters with excess presentations. Ignoring presentations prevents identification of clusters where more presentations, but not necessarily more individuals, are occurring than expected by chance. Surveillance of presentations to EDs can identify geographic areas with high presentations where access to other health care providers is limited and statistical detection of these areas necessitates incorporating repeated presentations by individuals.

There are several different tests for the identification of clusters of diseased individuals (cases) in geographic areas (see [1,2] for overviews). The geographic areas are generally administrative regions for which case and population counts are available. Besag and Newell [3] used the terms general and focused to distinguish between tests that identify clusters in a geographic region and those that identify clusters around specific geographic locations, such as hazardous waste sites. For both types of tests, the methods generally either examine areas with similar population size and compare the number of cases or examine areas with similar numbers of cases and compare the population sizes. Some methods can detect the location of clusters while others can identify areas with the tendency to cluster.

We focus on methods that can accommodate geographic areas with different population sizes. One exploratory method has been proposed by Openshaw et al. [4] and requires the user to specify a threshold. Their geographic analysis machine constructs overlapping circles and displays the circles with proportions of cases exceeding the threshold. The Turnbull et al. [5] method also uses overlapping circles but these circles require a user-specified population size. The maximum number of cases in each of the circles is determined and a Monte Carlo test assesses if the observed proportion is higher than expected. Kulldorff and Nagarwalla [6] generalize this approach and seek to identify a clustered circle, where the individuals inside the circle have greater risk than the individuals outside the circle, using a likelihood ratio test. Duczmal and Assunção [7] propose a similar approach that uses connected subgraphs to identify the cluster. Tango [8] takes a different strategy by using area proportions of cases and a measure of closeness. A chi-square test compares observed and expected proportions to detect clusters. Besag and Newell [3] look at similar numbers of cases and combine nearby areas to observe a user-specified number of cases. The population sizes of these areas with similar cases are then compared. More recently, Rosychuk et al. [9] have provided a similar method for events rather than cases by using a compound Poisson distribution and compared the two approaches [10].

Herein, we provide an approach to identify geographic areas with aggregations of events. These aggregations are called event clusters, or simply clusters when the distinction between aggregations of events and cases is clear. Our approach is analogous to the cluster detection test proposed by Besag and Newell [3] and does not require distributional assumptions, like the Rosychuk et al. [9] test. The approach is instead based on exact probabilities for the numbers of events in a tested geographic area. The method incorporates diverse population sizes and population distributions that can differ by important strata. This method requires the number of events per case is known. In addition, we provide an approach that can be used when the total number of events are known for the cases in a geographical area, but not the number of events per case. We describe the methodology and compare the methods using administrative data on emergency department (ED) presentations for self-inflicted injuries. We provide a simulation study that examines the false detection of clusters and conclude our discussion.

## Results

We consider a geographic region divided into *I *cells where the population in cell *i *is denoted by *n_i_*, *i *= 1, ..., *I*. The total population is labeled . The distances between cell centroids are calculated for each pair of cells. For each cell, the remaining cells are ordered based on increasing pairwise distances to determine the nearest neighbours. Specifically, we let *i*_1 _be the cell that is closest to cell *i*, *i*_2 _be the cell that is next closest to cell *i*, and so on. For convenience, we have *i*_0 _= *i*.

We first describe the Besag and Newell [3] approach for cases using a hypergeometric distribution. We provide our new exact method for events and review the compound Poisson approach developed by Rosychuk et al. [9]. We also describe the inclusion of strata variables and the choice of event cluster size.

### Hypergeometric approach for cases

The Besag and Newell [3] method requires the number of cases and population in each cell. For each cell *i*, let *C_i _*be the random variable denoting the number of cases in the cell and let *c_i _*be the observed value. Further, let  and  be the random variable and the observed value of the total number of cases in the entire region, respectively. In practical applications for a rare disease, *c_i _*≪ *n_i _*and generally *c *≪ *n_i_*, for *i *= 1, ..., *I*.

Each cell *i *is tested separately and the test is based on combining nearest neighbours to obtain a minimum pre-specified number of cases, called the cluster size. For cell *i*, suppose that the cluster size is *k*. The test statistic, *L_i_*, is the smallest number of nearest neighbours that must be combined with *i *to have at least *k *cases,

Smaller observed values of *L_i _*are indicative of higher numbers of cases in the vicinity of the tested cell.

Under a null hypotheses that all individuals are equally likely to be a case independent of other cases and geographic location, Besag and Newell [3] use a Poisson distribution as an approximation to the hypergeometric probability. Using the exact hypergeometric formulation, the probability that there are exactly *x *cases in a sample of *m *population is(1)

If we denote the population of the combined cells as , then the significance level for the tested cell becomes(2)

We will refer to this testing approach as the hypergeometric case (HC) analysis in further discussions.

As each cell is tested separately and multiple testing is an issue, Besag and Newell [3] suggest Monte Carlo simulations to help assess the significance of overall clustering. Let *R_α _*denote the number of cells that are significant at significance level *α*. For each simulation, the *c *cases are randomly distributed to the *I *cells according to the proportion of population in each cell (i.e., cases are distributed to cell *i *based on probability *n_i_*/*n*). The testing is conducted and the number of significant cells for each simulation is recorded. The overall assessment of statistical significance is obtained as the proportion of simulations that are at least as extreme as the observed *R_α _*from the actual data set. We refer to this overall assessment as a *p*-value for overall clustering.

### The proposed approaches for events

Our proposed method builds on the principles of the HC method and the approach of Rosychuk et al. [9] that concentrates on the number of disease-related events rather than the number of diseased individuals. As before, *C_i _*denotes the random variable corresponding to the number of cases in cell *i*. Let *V_i _*and *v_i _*be the random variable and observed value of the number of events in cell *i*, respectively. The total number of cases (i.e., individuals with at least one event) and events for the region are denoted by  and  with observed values  and , respectively. In most applications, *c_i _*<*v*_*i*_≪ *n_i _*and *c *<*v *≪ *n_i _*for *i *= 1, ..., *I*, but such restrictions are not required.

Each cell is tested separately as for the cases approach described above. The cluster size, now termed event cluster size, is the number of events rather than the number of cases used in the test. Let k* be the event cluster size for cell *i*. The test statistic is the smallest number of nearest neighbours that must be combined with cell *i *to have at least k* events,(3)

Smaller observed values of  are indicative of higher numbers of events in the vicinity of the tested cell.

The use of events requires changes to the null hypothesis and the exact probability. The null hypothesis is that every individual is equally likely to have events, independent of other individuals and geographic location. The hypergeometric probability is no longer appropriate if there is more than one event per case. If the number of events per case is known, then a multiple hypergeometric approach can be used as described below. If the number of events per case is unknown but the total number of cases and events are known, then a counting approach which employs occupancy numbers can be used.

### Known number of events per case

Let *C_iy _*be the random variable denoting the number of cases in cell *i *that have exactly *y *events and let *c_iy _*denote its observed value. Suppose that the maximum number of events any subject has is *Y*, 1 ≤ *Y*. The total number of cases in cell *i *is  and the total number of cases in the entire study region with exactly *y *events is . Further, the total number of cases is  For each cell *i*,  is the number of events in the cell.

The probability of the number of events observed in a geographic area is based on a multiple hypergeometric distribution, since individuals with the same number of events can be thought of as classes and the subjects are sampled without replacement from these classes. The probability of observing *x *events among a sample of *m *individuals is(4)

where {*r*_*y *_} are non-negative integers from the set ,

 = {(*r*_1_, ..., *r_Y_*) such that  and *r_y _*≤ *C*_•*y*_, *y *= 1, ..., *Y*} Note that if the maximum number of events per subject is one (*Y *= 1), then (4) simplifies to the hypergeometric distribution in (1). The significance level for the tested cell *i *becomes(5)

and we refer to this approach as the exact event (EE) analysis.

In practical application, the random variables representing events are replaced by the corresponding observed values and the expected number of events, *n_i:ℓ _v/n*, is helpful in describing the results. Simulated data sets are created analogously as described for the Besag and Newell approach for cases and a *p*-value for overall clustering is determined. We used a C^++ ^program for numerical results and *M *(*x, n_i:ℓ_*) is quite easily obtained by convoluting binomial coefficients and storing the intermediate quantities. The terms  and  are common to each combination of (*r*_1_, ..., *r_Y_*), and thus can be pre-computed and simplified for better numerical stability by using logarithms.

Our new approach is similar to the test provided by Rosychuk et al. [9]. The difference in approaches is the way in which the key probabilities are calculated. In that work [9], instead of the probability in (4), probabilities from a compound Poisson distribution are used. Specifically, *P *(*x, n_i:ℓ_*) replaces *M *(*x, n_i:ℓ_*) in (5) and is defined by(6)(7)

where *Q*(*y*) is the probability that a case has exactly *y *events. Those authors take *Q*(*y*) to be the observed frequency *c_•y_/c *and with this choice, the expected number of events becomes *n_i:ℓ _*(*c/n*) (*v/c*) = *n_i:ℓ _v/n*. This approach, referred to as the compound Poisson event (CPE), assumes that a compound Poisson distribution is appropriate and relies on estimates of the *Q*(*y*) that may not be very precise. Both methods have the same expected number of events but these events come from distinct distributions. As in the HC approach for cases, Monte Carlo simulations can be used to help assess overall clustering.

### Aggregate number of events known only

In some situations, the total number of events, *v_i _*and *v*, and cases may be known but the exact number of events per case (e.g., *c_iy_*, *c_•y_*) may not be known. Here, the relevant probability can be determined using a counting approach employing occupancy numbers (see [11] p. 38).

The number of ways that *V *events can be distributed among the *n *individuals is . From a sample population of size *m*,  is the number of ways that *x *events can be distributed among the *m *individuals and  is the number of ways to distribute the remaining *V *- *x *events among the remaining *n *- *m *individuals. Putting these counts together, the probability of observing *x *events among a sample of *m *individuals is(8)

The significance level for the tested cell becomes(9)

and we refer to this approach as the aggregate event (AE) analysis. As in the EE approach, random variables are replaced by the corresponding observed values and simulation is used to assess overall significance.

A key difference with this new aggregate event method and the EE and CPE methods is that the latter two methods link the events to cases. The AE approach uses only aggregate events, assumes that events are indistinguishable, and does not include information on the number of cases or the chance that a particular number of events are observed from one subject. Hence a loss of information occurs with the use of the AE approach, however if the information available does not include the number of events per case then the EE and CPE methods are not applicable and the AE method permits analysis.

### Stratification

Cells can have population distributions that differ on key characteristics such as gender and age. These strata can be added to the test to adjust for differing population distributions. Suppose there are *S *strata. The population in cell *i *and stratum *s *is *n_is_*, *s *= 1, ..., *S*, and relevant aggregate populations become  and . For stratum *s*, let *C_iys _*be the random variable denoting the number of cases in cell *i *that have exactly *y *events. The total number of cases in the entire study region in stratum *s *with exactly *y *events is . Similarly, *V_is _*is the number of events in cell *i *and stratum *s *and the number of events in cell *i *is 

The test statistic in (3) also applies when strata are added. The relevant probability is based on (4), but has to be modified to include the different strata and the multiple ways that the events may be distributed amongst the strata. The probability of observing *x *events among a sample of *m *= *m*_1 _+ *... *+ *m_S _*individuals is(10)

where = {(*r*_11_, ..., *r*_*Y*1_, ..., *r*_1*S*_, ..., *r*_*YS*_), such that  and *r_ys _= C_•ys_, y *= 1, ..., *Y, s *= 1, ..., *S*}. The significance level for the tested cell *i *becomes(11)

Where  is the population in cell *i *and its ℓ nearest neighbours that belong to stratum *s*, s = 1, ..., *S*.

The incorporation of strata tends to reduce the computational load as compared to the calculations without strata. The number of cases with events in a particular stratum is smaller than the total number of cases with events and thus, fewer combinations of (*r*_1*s*_, ..., *r*_*Ys*_) will be required. These combinations can then be calculated through a convolution.

The AE approach can be similarly extended for strata. The total number of events in stratum *s *is . The probability of observing *x *events among a sample of *m *= *m*_1 _+ *... *+ *m_S _*individuals is(12)

where  = {(*x*_1_, ..., *x_S_*) such that  and *x*_*s *_≤ *V*_•*s *_for *s *=1, ..., *S*}. The significance level for the tested cell becomes(13)

### Choosing k*

The actual size of a real cluster will not be known. The tests are based on identifying a cluster of a particular size, k*. With the dependence on k*, its choice is key and will depend on the specific data context. If the population sizes are similar among cells, a pre-specified, meaningful k* may be chosen and used for testing each cell. If population sizes are quite different, then cells may be better tested at individually-chosen, pre-specified k*. Le et al. [12] proposed a testing algorithm for the Poisson version of the HC method and a similar scheme is used for CPE approach [9]. We follow the latter approach.

In our prescription, event cluster sizes , are determined for cell *i*, *i *= 1, ..., *I*. Cell *i *is tested at  and if it is significant at a level *α*, testing is concluded for this cell. If it is not significant the cell is tested again at . The testing algorithm proceeds in this matter until the tested cell is significant at an event cluster size or the set of event cluster sizes has been exhausted. This approach is similar to sequential analysis and the event cluster sizes are defined as(14)

for tests of size *α*, *w *= 0, 1, 2. The event cluster sizes are based on the 100 × (1 - *α*) percentile of the distribution of events with populations from the cell and up to three of its nearest neighbours. ¿From this definition, cells with significant tests will have the test statistic equal to *w *whereas cells without sufficient events will have to combine more than *w *neighbours (i.e., *> w*). With potentially multiple event cluster sizes tested per cell, the Monte Carlo simulations are an important aspect to address the potentially increased testing. This approach to choosing the event cluster size can be easily extended to depend on strata and/or determined similarly for the AE approach.

We have chosen *w *to be at most 2 for our application because some of our geographic areas are quite large, either in population or geography, and *w *beyond 2 would potentially mean that large geographic areas and/or large population areas would be tested together. It is unlikely that such a situation would yield a statistically significant cluster, since the population sizes become quite large. Other choices for *w *are possible and would depend on the particular geography involved and the user's preference. In particular, one could potentially have different numbers of tests per cell (i.e., *w*_*i*_).

### Self-inflicted injury data

We use data from an administrative database that includes all presentations to emergency departments (EDs) in the province of Alberta, Canada. We focus on presentations to EDs for self-inflicted injuries by adolescents (≥ 13 and < 18 years of age) during April 1, 1998, to March 31, 1999. Individuals with at least one ED presentation during the study period are the cases and the ED presentations are the events. Data are stratified by sex (male, female) and age group (13-14, 15-17 years).

**Figure 1:**
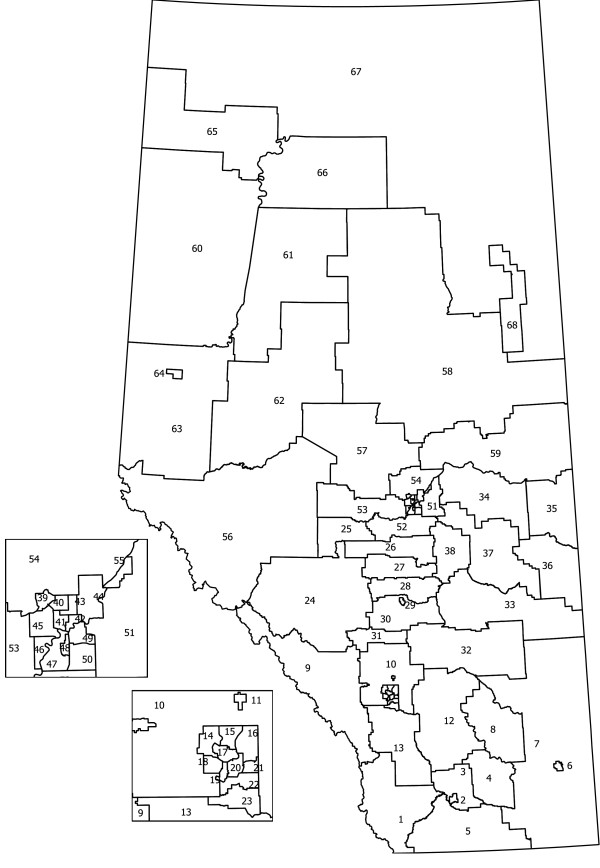
**Alberta sub-Regional Health Authorities (HAs)**.

The province of Alberta (Figure 1) is divided into *I *= 68 sub-Regional Health Authorities (HAs) with very diverse population sizes (median 2975, range 599 to 10298). During the study period, adolescents numbered *n *= 223999 in the population and *c *= 764 individuals presented *v *= 852 times to the ED with self-inflicted injuries. Although most individuals presented once, the range of presentations was one to 18. For the HAs, the median number of cases was 7.5 (range 0 to 43) and the median number of events was 8 (range 1 to 51). Alberta Health and Wellness provided the administrative data and the distances between population-based centroids. For each HA, Alberta Health and Wellness has determined a population-based centroid that is the latitude and longitude of the centre of the geographic area weighted by population. The distances between centroids are calculated and the ordering is provided for each cell.

Key to this illustrative example is that individuals can make multiple ED presentations during the study period. If analysis is based on the cases alone and not the presentations, then a case with only one presentation and a case with 10 presentations are treated exactly the same. The information on the "extra presentations" is ignored and only areas with excess number of cases can be identified as a cluster. Areas that have excess numbers of presentations, but not necessarily excess numbers of cases, cannot be identified unless the analysis includes the presentation information. From the health services perspective, the presentations are the most appropriate unit of analysis and while identifying areas with excess cases is important, the identification of excess areas of presentations may suggest areas where disease severity may be greater or alternative health care options are not as available.

**Figure 2:**
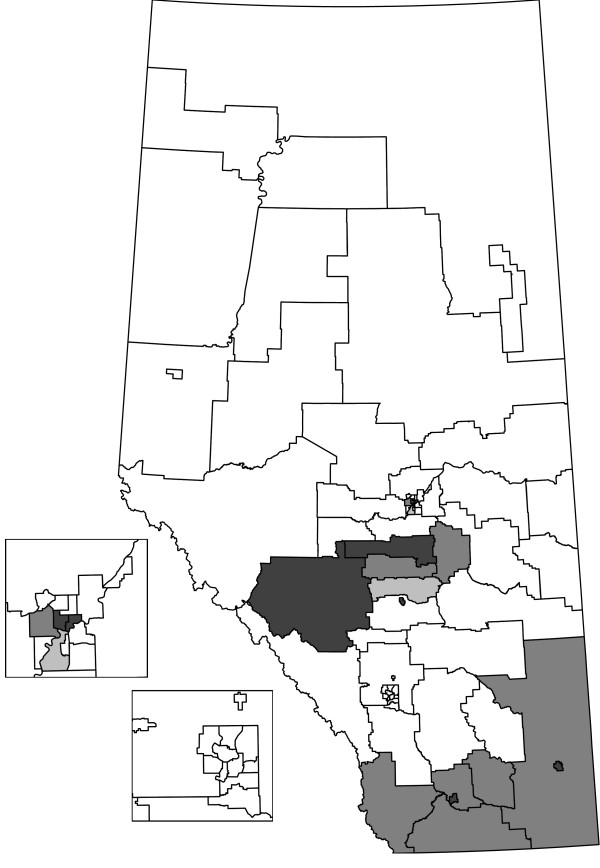
**Shaded HAs are significant as clusters or parts of clusters for the HC analysis**.

**Figure 3:**
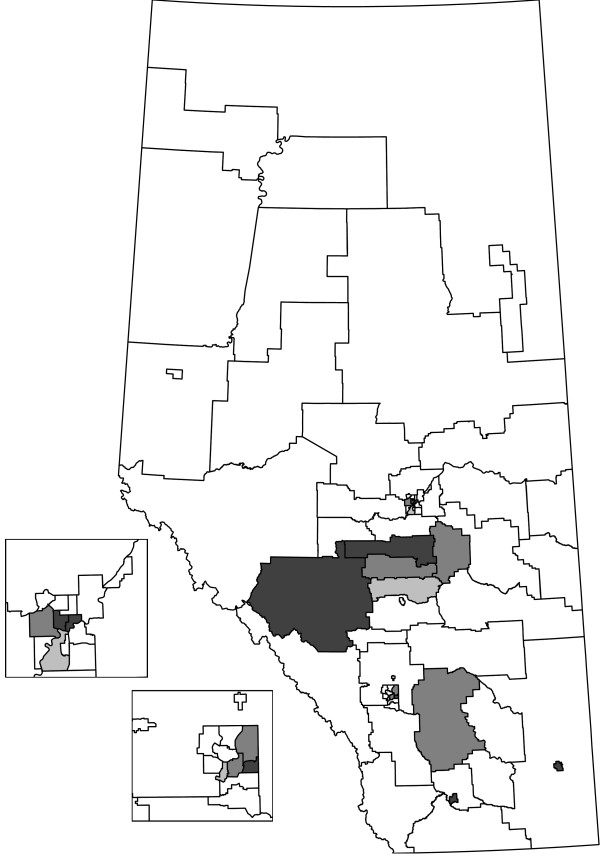
**Shaded HAs are significant as clusters or parts of clusters for the CPE analysis**.

**Figure 4:**
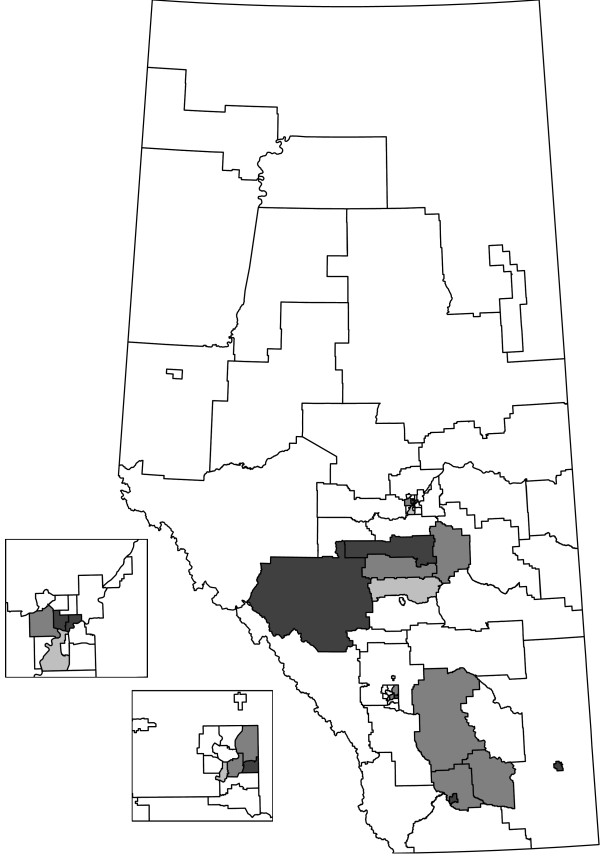
**Shaded HAs are significant as clusters or parts of clusters for the EE analysis**.

We compare the HC, CPE, and EE methods on the Alberta data set stratified by sex and age group (Table 1). The table lists the size of the cluster tested (*k *or k*), observed test statistic (*ℓ*), and the observed (*O*) and expected (*E*) cases or events. Tests that were significant at *α *= 0.05 are indicated with an asterisk (*). HAs that are not significant were tested at all cluster sizes, from *w *= 0 to 2, with the results for the last test reported. For the significant HAs, the *w *is the same as the value of *ℓ*. For non-significant HAs, the number of cells (*ℓ*) that need to be combined to have at least the size of the cluster tested is larger than *w*. The HAs are grouped according to the Regional Health Authority that provides services to the HAs (e.g., HAs 1 to 5 are part of the same Regional Health Authority). Figures 2, 3, and 4 display the HAs that are significant, either alone or in combination with other HAs. For each method, the simulation-based overall tests suggest that the results are not likely to have occurred by chance. In the 1000 simulations for the HC case analysis, 3 had at least 15 clusters observed (overall *p*-value = 0.003). For the event analyses, the CPE approach had 5 simulations with at least 13 clusters (overall *p*-value = 0.005) and the EE approach had 0 simulations with at least 15 clusters (overall *p*-value = 0.000).

**Table 1:** Clustering results for the Alberta adolescent self-inflicted injury data from each of the three approaches

Case Analysis										Event Analysis								
	
HC							CPE						EE					
***i***	***k***	***ℓ***	***O***	***E***	***O*/*E***	***p***	k*	***ℓ***	***O***	***E***	***O*/*E***	***p***	k*	**ℓ**	***O***	***E***	***O*/*E***	***p***

1	43	2	45	32.3	1.4	0.038*	51	3	51	42.6	1.2	0.154	51	3	51	42.6	1.2	0.150
2	24	0	31	16.2	1.9	0.039*	28	0	33	18.1	1.8	0.049*	28	0	33	18.1	1.8	0.048*
3	31	1	34	22.1	1.5	0.040*	44	3	50	40.9	1.2	0.332	43	2	43	30.0	1.4	0.049*
4	37	2	39	26.9	1.4	0.035*	44	3	50	40.9	1.2	0.332	43	2	43	30.0	1.4	0.049*
5	35	1	37	25.9	1.4	0.048*	50	3	50	40.9	1.2	0.134	50	3	50	40.9	1.2	0.130

6	20	0	21	12.9	1.6	0.038*	23	0	24	14.4	1.7	0.049*	23	0	24	14.4	1.7	0.048*
7	27	1	28	18.8	1.5	0.041*	41	3	41	33.3	1.2	0.143	41	3	41	33.3	1.2	0.140
8	26	5	43	42.8	1.0	0.998	31	5	48	47.7	1.0	0.989	31	5	48	47.7	1.0	0.991

9	55	5	66	82.9	0.8	1.000	65	5	77	92.4	0.8	0.996	64	5	77	92.4	0.8	0.998
10	35	4	71	77.4	0.9	1.000	41	4	80	86.3	0.9	1.000	41	4	80	86.3	0.9	1.000
11	72	3	78	76.0	1.0	0.705	85	3	91	84.8	1.1	0.479	84	3	91	84.8	1.1	0.518
12	47	3	69	70.6	1.0	0.999	36	1	42	24.0	1.8	0.046*	36	1	42	24.0	1.8	0.044*
13	90	4	97	94.9	1.0	0.722	106	4	126	105.8	1.2	0.483	105	4	126	105.8	1.2	0.519
14	69	6	111	123.6	0.9	1.000	81	6	130	137.8	0.9	1.000	81	6	130	137.8	0.9	1.000
15	84	3	92	95.1	1.0	0.901	98	3	108	106.1	1.0	0.737	97	3	108	106.1	1.0	0.780
16	73	3	78	76.8	1.0	0.695	72	1	89	54.6	1.6	0.050*	72	1	89	54.6	1.6	0.047*
17	60	4	63	83.8	0.8	0.998	71	4	73	93.4	0.8	0.981	70	4	73	93.4	0.8	0.990
18	46	3	49	61.2	0.8	0.985	55	3	59	68.2	0.9	0.921	54	3	59	68.2	0.9	0.945
19	38	3	46	54.5	0.8	0.994	46	3	52	60.8	0.9	0.957	45	3	52	60.8	0.9	0.972
20	40	3	53	56.2	0.9	0.992	48	2	49	33.6	1.5	0.044*	48	2	49	33.6	1.5	0.043*
21	73	3	91	85.5	1.1	0.935	26	0	38	16.1	2.4	0.042*	26	0	38	16.1	2.4	0.041*
22	80	3	80	76.4	1.0	0.351	94	4	125	101.3	1.2	0.720	93	4	125	101.3	1.2	0.763
23	80	3	80	76.4	1.0	0.351	94	4	125	101.3	1.2	0.720	93	4	125	101.3	1.2	0.763

24	11	0	13	5.8	2.2	0.035*	13	0	16	6.5	2.5	0.035*	13	0	16	6.5	2.5	0.034*
25	44	5	71	61.3	1.2	0.993	53	5	80	68.4	1.2	0.952	52	5	80	68.4	1.2	0.968
26	17	0	28	10.5	2.7	0.040*	20	0	31	11.8	2.6	0.043*	20	0	31	11.8	2.6	0.042*
27	32	2	36	23.2	1.5	0.046*	38	2	39	25.9	1.5	0.049*	38	2	39	25.9	1.5	0.048*
28	40	3	43	39.6	1.1	0.499	48	4	74	56.0	1.3	0.819	48	4	74	56.0	1.3	0.828
29	26	0	28	17.5	1.6	0.032*	54	5	61	55.2	1.1	0.542	54	5	61	55.2	1.1	0.545

30	41	3	42	38.7	1.1	0.378	49	5	51	65.0	0.8	0.964	49	5	51	65.0	0.8	0.969
31	38	3	43	45.6	0.9	0.896	45	4	49	58.4	0.8	0.943	45	4	49	58.4	0.8	0.949
32	37	6	38	54.7	0.7	0.996	44	7	66	80.5	0.8	1.000	44	7	66	80.5	0.8	1.000
33	27	4	29	29.7	1.0	0.722	32	5	35	41.8	0.8	0.915	32	5	35	41.8	0.8	0.921

34	27	3	29	35.6	0.8	0.946	32	4	34	44.3	0.8	0.956	32	4	34	44.3	0.8	0.961
35	25	4	34	40.3	0.8	0.997	30	4	36	44.9	0.8	0.982	30	4	36	44.9	0.8	0.984
36	26	5	26	35.5	0.7	0.962	31	6	45	58.6	0.8	1.000	30	6	45	58.6	0.8	1.000
37	27	6	54	46.0	1.2	0.999	32	6	59	51.3	1.1	0.995	32	6	59	51.3	1.1	0.996
38	25	1	32	17.5	1.8	0.049*	30	1	36	19.5	1.9	0.047*	30	1	36	19.5	1.9	0.046*

39	53	3	55	51.7	1.1	0.443	63	4	74	75.6	1.0	0.895	63	4	74	75.6	1.0	0.906
40	53	3	55	51.7	1.1	0.443	63	4	76	69.6	1.1	0.743	63	4	76	69.6	1.1	0.753
41	16	0	21	9.9	2.1	0.045*	19	0	22	11.1	2.0	0.043*	19	0	22	11.1	2.0	0.042*
42	17	0	19	10.8	1.8	0.046*	20	0	20	12.0	1.7	0.047*	20	0	20	12.0	1.7	0.046*
43	56	3	61	54.8	1.1	0.453	66	4	85	72.2	1.2	0.723	66	4	85	72.2	1.2	0.734
44	66	3	73	63.9	1.1	0.409	78	4	79	82.2	1.0	0.639	77	4	79	82.2	1.0	0.685
45	50	2	58	39.2	1.5	0.049*	60	2	62	43.7	1.4	0.045*	60	2	62	43.7	1.4	0.043*
46	51	3	54	50.4	1.1	0.484	60	3	60	56.2	1.1	0.327	60	3	60	56.2	1.1	0.324
47	45	3	54	50.4	1.1	0.803	53	3	60	56.2	1.1	0.628	53	3	60	56.2	1.1	0.634
48	44	3	59	61.6	1.0	0.994	53	3	68	68.7	1.0	0.955	52	3	68	68.7	1.0	0.971
49	42	3	59	59.2	1.0	0.994	50	3	66	66.0	1.0	0.962	49	3	66	66.0	1.0	0.976
50	61	4	78	72.3	1.1	0.932	72	4	88	80.7	1.1	0.789	71	4	88	80.7	1.1	0.830
51	61	4	61	67.1	0.9	0.800	72	5	82	86.8	0.9	0.916	71	5	82	86.8	0.9	0.941
52	62	4	68	73.3	0.9	0.929	73	4	77	81.7	0.9	0.788	72	4	77	81.7	0.9	0.829
53	57	4	74	74.3	1.0	0.988	68	4	80	82.9	1.0	0.923	68	4	80	82.9	1.0	0.933
54	46	4	67	69.3	1.0	0.999	55	4	69	77.3	0.9	0.990	55	4	69	77.3	0.9	0.993
55	54	3	54	52.4	1.0	0.427	64	4	68	74.6	0.9	0.853	64	4	68	74.6	0.9	0.864

56	31	3	37	28.2	1.3	0.324	37	3	43	31.5	1.4	0.207	37	3	43	31.5	1.4	0.204
57	52	4	54	67.8	0.8	0.984	62	5	71	91.7	0.8	0.998	61	5	71	91.7	0.8	0.999
58	43	4	50	54.0	0.9	0.952	51	4	54	60.2	0.9	0.847	51	4	54	60.2	0.9	0.857
59	44	4	54	58.6	0.9	0.983	53	4	56	65.3	0.9	0.910	53	4	56	65.3	0.9	0.919

60	33	6	33	42.7	0.8	0.950	40	8	51	64.2	0.8	0.998	40	8	51	64.2	0.8	0.999
61	27	3	29	30.6	0.9	0.770	32	4	34	42.4	0.8	0.927	32	4	34	42.4	0.8	0.932
62	32	4	32	38.0	0.8	0.862	38	5	48	55.5	0.9	0.986	38	5	48	55.5	0.9	0.988
63	36	5	44	49.8	0.9	0.986	43	5	48	55.5	0.9	0.935	43	5	48	55.5	0.9	0.941
64	35	5	44	49.8	0.9	0.991	41	5	48	55.5	0.9	0.963	41	5	48	55.5	0.9	0.967

65	14	4	15	20.4	0.7	0.947	16	5	25	29.7	0.8	0.994	16	5	25	29.7	0.8	0.995
66	14	4	15	20.4	0.7	0.947	16	5	25	29.7	0.8	0.994	16	5	25	29.7	0.8	0.995
67	14	4	15	20.4	0.7	0.947	16	5	34	36.1	0.9	1.000	16	5	34	36.1	0.9	1.000
68	52	4	57	55.3	1.0	0.696	61	4	62	61.6	1.0	0.513	61	4	62	61.6	1.0	0.515

The number of each HA (*i*) is provided along with the size of the cluster tested (*k *or k*), observed test statistic (*3*), the observed (*O*) and expected (*E*) cases or events, and the unadjusted *p*-value (*p*). An asterisk (*) denotes test significant at *a *= 0.05, unadjusted for multiple testing.

The HC and EE methods both identify 15 significant HAs as part of clusters, although the significant HAs are not the same in each analysis. The CPE method identifies 13 significant HAs, all of which are identified by the EE method. Generally, the HAs that were significant with all three methods had higher numbers of cases and events than expected by chance. Similarly, the HAs that were not found to be significant by any of the methods had fewer cases and events than expected by chance. The event cluster sizes are generally very close for the EE and CPE analyses, with the EE analyses having slightly lower sizes for some HAs. One thousand Monte Carlo simulations were done for each method and less than six simulated data sets in each approach were at least as extreme as the actual number of clusters observed. Hence, not all clusters are likely to be spurious.

We focus our discussion on the HAs where the methods have incongruous results. HAs 1, 5, 7, and 29 are significant as parts of clusters for the HC analysis, but not significant for the CPE and EE analyses. For these HAs, generally the number of cases is high and the number of events are just a bit lower than the CPE and EE cluster sizes. To be significant as a cluster alone in the CPE or EE analyses, HA 29 needed to have at least 30 events whereas it only had 28 events. HAs 3 and 4 were identified as clusters in the EE analysis, but not in the CPE analysis. In particular, the event cluster size required for the EE analysis was 43 based on the combination of HAs 3, 4, and 2. There were exactly 43 events in these combined cells and the EE test was significant. For the CPE analysis, the corresponding event cluster size was 44 and the combined HAs had too few events.

The fact that all of the significant HAs in the CPE analysis were all identified by the EE approach is not a feature that is likely to occur in all data sets. In particular, our event cluster sizes are relatively large and the relevant probabilities of the CPE and EE may be closer than in other situations. In addition, the compound Poisson distribution may not be an appropriate distribution for other data. Furthermore, our example had key HAs that were significant alone and when these HAs were combined with some other HAs, they too became significant. Typically, there would be some cells where the cells individually are not significant but when combined together do become significant.

In practice, a researcher would probably conduct both analyses based on cases and analyses based on events. Each analysis tests different aspects of data based on multiple disease-related events. If the median number of events is larger than one, then the analysis based on events is likely more informative. We would expect that users would prefer the EE approach over the CPE approach because it does not require distributional (parametric) assumptions, even if there is some power loss over parametric procedures. In addition, this approach can be very efficiently programmed even when there are strata variables. If there are no strata variables, then the CPE approach may be more timely.

### Simulation

We examine the Type I Error of the EE and CPE approaches through simulation studies. In these studies, the Alberta cells and geographic relationship are used. The cell populations are set to be the Alberta population or the same population in each cell (1000, 5000, or 8000). Five settings for the probability of multiple events per case are considered (Table 2) with varying means and skewness chosen for convenience. Setting S5 is based on the self-inflicted injury data in Alberta. The event rate is set to be 2 events per 1000 population. That is, the total number of events in each simulated data set was 136, 680, and 1088 for the settings with 1000, 5000, and 8000 population per cell, respectively. When the actual Alberta population is used, the total number of events is 447. With multiple event probabilities and crude event rates, the simulated data sets are created by randomly assigning the *c*_•*1*_, c_•*2*_, ... cases to the cells based on each cell's proportion of the total population (as in the Monte Carlo simulations for assessing overall clustering). These settings allow us to demonstrate that the detection of events is different than the detection of cases. In particular, clusters of events may be identified that are not also clusters of cases.

**Table 2:** Event probabilities for the simulation scenarios

Scenario	Non-zero Event Probabilities *Q*(*y*)				
S1	*Q*(1) = 0.6,	*Q*(2) = 0.3,	*Q*(3) = 0.1		

S2	*Q*(1) = 0.94,	*Q*(2) = 0.05,	*Q*(3) = 0.01		

S3	*Q*(1) = 0.8,	*Q*(2) = 0.1,	*Q*(3) = 0.05,	*Q*(4) = 0.03,	*Q*(5) = 0.01,
	*Q*(6) = 0.01				

S4	*Q*(1) = 0.8,	*Q*(2) = 0.15,	*Q*(3) = 0.05		

S5	*Q*(1) = 0.929,	*Q*(2) = 0.058,	*Q*(3) = 0.008,	*Q*(4) = 0.001,	*Q*(5) = 0.001,
	*Q*(9) = 0.001,	*Q*(18) = 0.001			

For each simulation setting, we generated 1000 data sets and applied the CPE and EE approaches to each data set. To make comparisons easier, we obtained the cluster sizes  for each approach and tested each cell only once. The results of the simulations are shown in Table 3. With the discreteness of the distributions, the cluster size may coincide with a smaller significance level than the significance level setting of *α *= 0.05. We provide the effective significance level, α_*_, for each scenario based on the cluster size and provide the number of simulations that had at least one cluster detected. For the scenarios with constant cell populations the α_* _is the same for each simulation whereas for the data sets based on the Alberta population, the mean and standard deviation (SD) for α_* _are provided. As each simulated data set has different testing results, the mean and SD for the number of significant clusters are also given.

**Table 3:** Simulation results for each cell size and scenario

		EE		CPE	
*n_i_*	Scenario	α_* _(SD)	Sim(SD)	α_* _(SD)	Sim(SD)
Alberta	S1	3.9(0.6)	4.0 (0.9)	3.9(0.6)	3.9 (0.9)
	S2	3.7(0.7)	3.7 (1.0)	3.7(0.7)	3.6 (1.0)
	S3	4.0(0.6)	4.0 (0.8)	4.0(0.6)	3.9 (0.8)
	S4	3.7(0.7)	3.6 (0.9)	3.7(0.6)	3.5 (0.8)
	S5	4.2(0.6)	4.1 (0.8)	4.2(0.6)	4.1 (0.8)

1000	S1	2.5	2.6 (0.5)	2.6	2.6 (0.5)
	S2	2.3	2.4 (0.5)	2.4	2.4 (0.5)
	S3	3.7	3.7 (0.6)	3.8	3.7 (0.6)
	S4	3.9	3.9 (0.6)	4.0	3.9 (0.6)
	S5	2.4	2.3 (0.5)	2.5	2.3 (0.5)

5000	S1	4.9	5.0 (0.7)	3.4	3.3 (0.5)
	S2	3.5	3.6 (0.6)	3.7	3.6 (0.6)
	S3	4.3	4.3 (0.6)	4.4	4.3 (0.6)
	S4	3.5	3.5 (0.5)	3.7	3.5 (0.5)
	S5	4.0	3.9 (0.5)	4.1	3.9 (0.5)

8000	S1	4.7	4.6 (0.7)	4.8	4.6 (0.7)
	S2	4.6	4.6 (0.6)	4.8	4.6 (0.6)
	S3	4.3	4.4 (0.7)	4.4	4.4 (0.7)
	S4	4.8	4.9 (0.7)	5.0	4.9 (0.7)
	S5	4.7	4.7 (0.7)	4.7	4.7 (0.7)

The effective significance level, α_*_, is provided for each scenario and each approach (and standard deviations [SDs] for the Alberta scenarios). The mean number of significant cells (Sim) are provide along with SDs. Numbers are given as percentages (%).

In general, the EE and CPE approaches have similar results and the results of each approach are close to the (effective) significance level. The CPE results tend to identify slightly fewer clusters than expected. With larger population sizes, the effective significance level is closer to 0.05. When the diverse population sizes of Alberta are used, the number of clusters detected is more variable. Both methods provide detection rates of false clusters that are close to what is expected by the (effective) significance level.

## Conclusions

We have provided a new method for the detection of aggregations of events related to diseased individuals, called event clusters. This method builds on the approaches of Besag and Newell [3] for cases and of Rosychuk et al. [9] for events. Our approach uses the exact probability of observing events from a sample of the population. It is applicable to situations where cases may have multiple events and the number of events are of interest. The population sizes can differ from cell to cell and can be adjusted by strata. The method is easy to implement in computer code and requires a minimal amount of data from the administrative region. We used a testing algorithm similar to sequential testing to determine the size of clusters specific to each cell and compared the new method with two other approaches using data on presentations to emergency departments for self-inflicted injuries. In some contexts, like our emergency department presentations, the number of events can be more relevant than the number of cases and new method provides an exact approach for determining aggregations of these events. The use of cases only does not necessarily capture the relevant type of clustering and analyses based on events can also complement analyses based on cases.

Our approach is based on exact probabilities and does not require distribution assumptions as in the compound Poisson approach by Rosychuk et al. [9]. Those authors had to specify a probability distribution for the situation when a case has exactly *y *events and use estimates in their application. The distribution could be misspecified, estimates may not be very precise, and the *p*-value does not capture the additional uncertainty of these estimates. Our method uses the multiple hypergeometric distribution that does require a distribution, or estimates, for the event probabilities. Our new approach is particularly appealing when analyses are conducted by strata variables. With more strata variables, there are fewer possible combinations to calculate and the use of convolutions makes the computational time less than that required by the compound Poisson approach.

One drawback of the testing algorithm is that the user must choose the number of cluster sizes to be tested. This choice is perhaps easier than choosing a particular cluster size to test, especially if areas have diverse population sizes. Furthermore, the structure of the algorithm dictates that statistically significant cells will have the unappealing feature that p-values will be close to the significance level. Additionally, overall clustering can be assessed through Monte Carlo simulations and the same approach could be used to examine individual cell tests (i.e., calculate the proportion of simulations for which a particular cell was statistically significant as described in Rosychuk et al. [9]).

Both our new method and the compound Poisson method require that the number of events per cases is known. We also introduced an approach for the situation when the number of cases and events are known, but not the number of events for each case. Administrative data sources may not record the number of events per case and we provided an analysis approach for this context. This approach considers events to be indistinguishable and does not specifically use the number of cases. While not preferable to the analysis with information on the events per case, this approach is applicable for less detailed administrative data.

Further work is necessary to compare the power of the different approaches to detect different sized clusters and to consider different data generating mechanisms. Most cases in our data example only had one event. Our event results were different than the case analysis but further examination should include larger numbers of events per case. Such investigations will give users a greater ability to decide which method is most appropriate for the surveillance of disease-related events in their jurisdiction.

## Competing interests

The authors declare that they have no competing interests.

## Authors' contributions

RJR developed created the methodology, performed the analysis, and wrote the initial draft of the manuscript. JLS developed and coded the efficient algorithm for all computations and revised the manuscript. All authors read and approved the final manuscript.
